# Predicting Pathological Response to Preoperative Chemotherapy in Pancreatic Ductal Adenocarcinoma Using Post-Chemotherapy Computed Tomography Radiomics

**DOI:** 10.7759/cureus.52193

**Published:** 2024-01-13

**Authors:** Shinichi Ikuta, Tsukasa Aihara, Takayoshi Nakajima, Naoki Yamanaka

**Affiliations:** 1 Department of Surgery, Meiwa Hospital, Nishinomiya, JPN

**Keywords:** machine learning, radiomics, neoadjuvant, pdac, pancreatic ductal adenocarcinoma

## Abstract

Introduction: Assessing the response to preoperative treatment in pancreatic cancer provides valuable information for guiding subsequent treatment strategies. The present study aims to develop and validate a computed tomography (CT) radiomics-based machine learning (ML) model for predicting pathological response (PR) to preoperative chemotherapy in pancreatic ductal adenocarcinoma (PDAC).

Methods: Retrospective data were analyzed from 86 PDAC patients undergoing neoadjuvant or conversion chemotherapy followed by surgical resection from January 2018 to May 2023. The cohort was randomly divided into training (70%, n = 60) and testing (30%, n = 26) sets. Favorable PR was defined as Evans grade IIb or greater. Radiomic features were extracted from post-chemotherapy CT images, and dimensionality reduction was performed using the least absolute shrinkage and selection operator (LASSO) logistic regression. Four ML classifiers (Light Gradient Boosting Machine (LGBM), Random Forest, AdaBoost, and Quadratic Discriminant Analysis) were evaluated for predicting a favorable PR. Model performance was primarily assessed using the area under the receiver operating characteristic curve (AUC), Brier score, and decision curve analysis.

Results: Forty-one (47.7%) patients had a favorable PR. LASSO analysis on the training set identified five radiomic features. The LGBM model demonstrated the best performance, with a training AUC of 0.902 and a testing AUC of 0.923. It also exhibited the lowest Brier scores, both in training (0.136) and testing (0.135). Decision curve analysis further confirmed its clinical potential.

Conclusion: The CT radiomics-based ML model exhibited promising performance in predicting PR in PDAC after neoadjuvant/conversion chemotherapy. This suggests clinical utility in optimizing surgical candidates and timing of surgery, leading to personalized treatment strategies.

## Introduction

Pancreatic cancer is a growing global concern, ranking seventh in cancer-related deaths worldwide [1]. The incidence is rising over the last 20 years, and projections suggest it could become the second leading cause of cancer deaths in the United States by 2030 [2]. Pancreatic ductal adenocarcinoma (PDAC), the most common subtype comprising more than 90% of cases, is characterized by its high malignancy and late diagnosis, resulting in a five-year survival rate of around 10% [3].

Surgical resection remains the preferred treatment option for localized PDAC because it offers the only chance for a cure. Recently, the management of PDAC has shifted towards the use of neoadjuvant chemo(radio)therapy especially in resectable and borderline resectable disease, as indicated by clinical trials [4-6]. This approach has demonstrated benefits, including an increase in margin-negative resection rates, a reduction in the rate of lymph node involvement, and an improvement in prognosis [4-6]. Furthermore, a subset of patients with initially unresectable disease underwent conversion surgery after a favorable response to chemotherapy, potentially leading to extended survival compared to those treated with non-surgical treatment alone [7].

The clinical challenge in preoperative chemotherapy for PDAC lies in the varied treatment responses due to its diverse biological behavior. An accurate assessment of treatment response is crucial for guiding management and optimizing outcomes. Multiple methods have been used to assess response to neoadjuvant/conversion chemotherapy, including pathological, radiological, and tumor marker responses. Endoscopic ultrasound-fine needle aspiration allows for direct sampling of tumor tissue but is a relatively invasive tool that has a risk of complications, such as needle tract seeding [8]. Contrast-enhanced computed tomography (CT), the most frequently used imaging modality for the local assessment of PDAC, presents challenges in distinguishing between viable tumors and treatment-related alterations in the restaging setting after chemo(radio)therapy [9,10]. For instance, despite significant treatment response at the histologic level, there might be no appreciable change in size or vascular involvement in CT [9,10]. Fluorine-18 fluorodeoxyglucose (FDG)-positron emission tomography (PET) is a functional imaging modality capable of detecting changes in tissue metabolism. While FDG-PET has been explored in PDAC for assessing residual viable cancer or treatment response [11], the associated high radiation exposure and cost can pose a significant burden on patients. Furthermore, the widely used tumor marker of PDAC, serum carbohydrate antigen (CA) 19-9 levels, lacks sensitivity and is insufficient as a sole biomarker for assessing treatment response [12]. Thus, the development of less invasive and reliable methods for assessing treatment response is a priority in the management of patients with PDAC receiving preoperative chemotherapy.

In recent years, the integration of radiomics and artificial intelligence into medical practice has marked a significant advancement. Radiomics involves extracting numerous quantitative features from imaging modalities, such as CT scans, enabling a comprehensive analysis that extends beyond the capabilities of the human eye [13]. The synergy of radiomics with machine learning (ML) holds the potential to further improve the performance of image-based prediction tasks [13]. Previous studies have demonstrated an association between CT-derived radiomic features and survival in several kinds of cancers including PDAC [14-16]. While the pathological treatment effect of resected PDAC has prognostic implications [17-20], few studies have explored the radiomics-based ML model to evaluate the pathological response (PR) of PDAC after preoperative chemotherapy. This study aimed to develop and validate an ML model based on radiomic features extracted from post-chemotherapy CT images for predicting the PR of PDAC preoperatively.

## Materials and methods

Study population and clinical data collection

A total of 173 patients with pancreatic cancer who underwent pancreatectomy at our hospital between January 2018 and May 2023 were retrospectively identified. Of these, patients meeting the following inclusion criteria were included: (i) those receiving neoadjuvant chemotherapy for resectable or borderline resectable diseases, (ii) those receiving conversion chemotherapy for unresectable disease, (iii) those undergoing proximal, distal, or total pancreatectomy with lymphadenectomy, and (iv) those requiring pancreatectomy combined with other organ resections (e.g., liver resection, right hemicolectomy) or vascular resection and reconstruction. Exclusion criteria included: (i) lack of a pathological diagnosis of PDAC, (ii) those undergoing upfront surgery, (iii) absence of preoperative contrast-enhanced CT within a month before surgery, (iv) receipt of radiotherapy with chemotherapy, (v) not well-visualized pancreatic tumors on post-chemotherapy CT due to isoenhancement and inconspicuity, and (vi) missing or incomplete clinical data. Consequently, 86 patients were enrolled and analyzed.

Patient demographic and clinical data, including age, sex, body mass index, tumor location, resectability status, type of pancreatectomy, pre- and post-chemotherapy serum CA 19-9 levels, chemotherapy regimen, duration of therapy, histologic type, PR categorized by Evans grade, ypT stage, and ypN stage, were retrieved from electronic medical records. The resectability status was determined at the time of diagnosis based on the criteria outlined in the National Comprehensive Cancer Network guideline (ver. 1.2019) [21]. Evans grading for the primary pancreatic tumor was categorized by an expert pathologist as follows: (i) Evans grade I, little (< 10%) or no tumor cell destruction; (ii) grade IIa, destruction of 10-50% of tumor cells; (iii) grade IIb, destruction of 51-90% of tumor cells; (iv) grade III, < 10% viable-appearing tumor cells; (v) grade IV, no viable tumor cells [20]. In this study, Evans grades IIb, III, and IV were defined as favorable PR. The seventh edition of the American Joint Committee on Cancer manual was used for tumor staging [22].

Preoperative chemotherapy

The choice of chemotherapy regimen was not solely determined by resectability status but also depended on the initial specialty of the treating department at our hospital, either surgery or medical oncology. For a majority of patients with borderline resectable or unresectable PDAC, the GnPO-ITC regimen, consisting of nab-paclitaxel (125 mg/m^2^), gemcitabine (1,000 mg/m^2^), and oxaliplatin (85 mg/m^2^) on day 1, along with itraconazole (400 mg/day) on days -2 to +2 (UMIN-CTR: UMIN 000025398), was administered every two weeks within the medical oncology department for approximately six months [23]. In the surgical department, the gemcitabine plus S-1 regimen was predominantly used for resectable PDAC patients, typically administered over 1.5 months [4]. For patients with borderline resectable or unresectable disease in the surgical department, the primary course of treatment involved the FOLFIRINOX (a combination of oxaliplatin, irinotecan, fluorouracil, and leucovorin) or gemcitabine plus nab-paclitaxel regimen, given for approximately three months. For patients with unresectable disease with distant metastases, surgery was considered based on the following criteria: (i) a stable or improved primary tumor response according to the Response Evaluation Criteria in Solid Tumors criteria [24]; (ii) significant shrinkage or disappearance of metastases without the emergence of new lesions; (iii) reduction in serum CA 19-9 levels; (iv) decrease in the standardized uptake value by FDG-PET; and (v) sustained performance status and adequate organ function. Patients with residual metastatic disease after conversion chemotherapy were considered for surgery if all visible lesions were deemed potentially operable, regardless of the size, number, or location of the tumors.

CT image acquisition and tumor segmentation

Multiphasic CT scans (precontrast, arterial at 40 seconds, portal venous at 70 seconds, and equilibrium phase at 180 seconds) were routinely acquired using a Brilliance-iCT multidetector-row scanner (Koninklijke Philips N.V., Amsterdam, Netherlands) with scan parameters set at 120 kVp/200 mAs, 128×0.625-mm slice collimation, and 512×512 pixels. Images were typically reconstructed into 5-mm sections by radiology technologists and sent to the picture archiving and communication system (PACS) (ShadeQuest/ViewR ver. 1.30.10, Fujifilm Holdings Corporation, Tokyo, Japan) for interpretation. Axial CT images with pancreatic tumors, obtained within a month before surgery, were retrieved in digital imaging and communications in medicine format from the PACS and imported into an open-source software package (ITK-SNAP ver. 4.0.1) for segmentation. Contrast standardization was achieved with a window level of 40 Hounsfield units (HU) and a window width of 350 HU. An experienced radiologist, blinded to lesion outcomes, independently manually segmented all lesions, using the portal venous phase for its consistent tumor visualization and background tissue enhancement across cohorts.

Radiomic feature extraction and selection

The segmented data underwent analysis using Pyradiomics (ver. 3.0.1), an open-source Python (ver. 3.11.1) package, resulting in the extraction of a comprehensive set of radiomic features. These features encompass first-order statistics, three-dimensional (3D) shape-based features, gray-level co-occurrence matrix, gray-level dependence matrix (GLDM), gray-level run-length matrix, gray-level size zone matrix (GLSZM), and neighboring gray-tone difference matrix. To enhance model efficiency and reduce dimensionality, features were selected using the least absolute shrinkage and selection operator (LASSO) logistic regression. The regularization parameter (alpha) was tuned through a five-fold cross-validation process to minimize the mean squared error. Subsequently, the identified radiomic features were incorporated into the construction of the ML model.

ML model construction and validation

**Figure 1:**
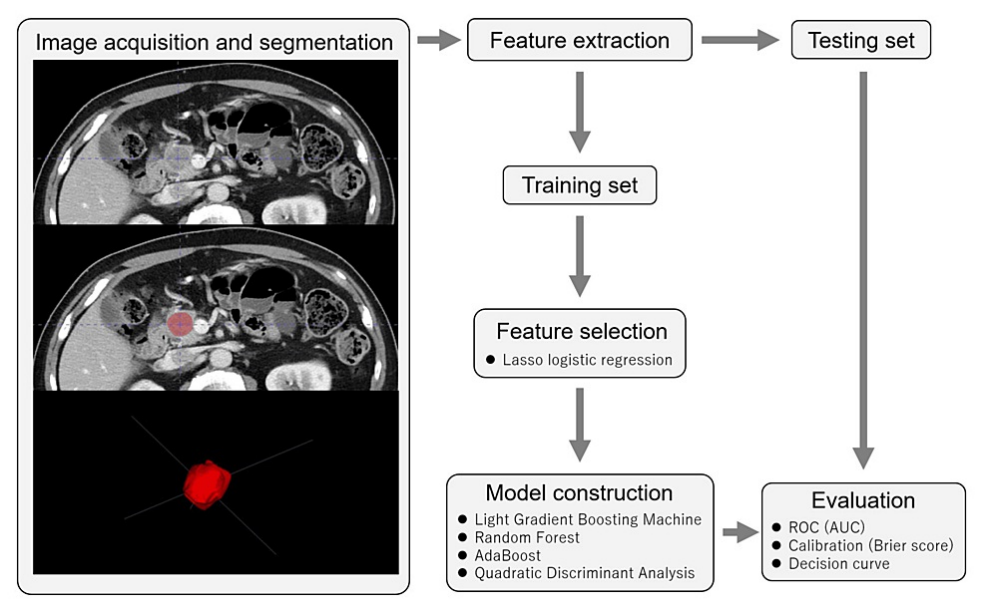
Flowchart illustrating the image processing, radiomic feature extraction, and machine learning workflow ROC, Receiver operating characteristic curve; AUC, Area under the curve

The ML model was constructed using PyCaret (ver. 3.0.0), an automated ML library in Python designed for streamlined processes with minimal code. Input values consisted of selected radiomic features, and output values included PR information (Evans grade IIb or greater). We evaluated four ML classifiers: Light Gradient Boosting Machine (LGBM), Random Forest (RF), AdaBoost (AB), and Quadratic Discriminant Analysis (QDA). Each classifier underwent 10-fold cross-validation, followed by hyperparameter tuning using default automated features, including random grid search, to optimize and construct the final model. To validate the model's robustness, a 30% holdout testing set was employed. While the primary evaluation centered on area under the receiver operating characteristic curve (AUC), Brier score, and decision curve analysis (DCA), we also assessed accuracy, sensitivity, specificity, and f1 score for a comprehensive performance analysis. The Brier score, which ranges from 0 to 1, assesses the squared differences between predicted and observed outcomes, with a lower score indicating superior performance. DCA was performed by calculating the net benefits for a range of threshold probabilities. The f1 score was calculated as follows: f1 score = 2 × (sensitivity × positive predictive value)/(sensitivity + positive predictive value). The process of image processing, radiomic feature extraction, and ML is shown in Figure 1.

Data analysis

No sample size calculations were performed in the present study. Continuous variables were summarized using median and range; categorical variables were summarized using frequency and percentages. When classifying patients into those with and without a favorable PR, differences between the groups were assessed using Fisher's exact test for categorical variables, and the Mann-Whitney U-test for continuous variables. These analyses were conducted using SciPy (ver. 1.10.1) in Python, and a p-value of less than 0.05 was considered statistically significant. In addition to the aforementioned packages, the following packages were used for data analyses and visualization: Pandas (ver.1.5.3), NumPy (ver. 1.23.5), scikit-learn (ver. 1.2.2), and Matplotlib (ver. 3.7.1).

## Results

Clinical characteristics

The study cohort comprised 45 men and 41 women with a median age of 66 years (range: 39-83 years). Among them, 41 (47.7%) showed a PR with 25 having Evans grade IIb, 16 having grade III, and none having grade IV. Patients were randomly stratified into a training set (n = 60) and a testing set (n = 26), maintaining a balanced distribution of favorable PR, with 29 (48.3%) in the training set and 12 (46.2%) in the testing set. Patient characteristics are shown in Table 1. Patients with favorable PR showed a higher prevalence of ypT1/T2 compared to those without in both the training and testing sets. Moreover, a significant increase in ypN0 was observed among those with favorable PR within the testing set. We observed no statistically significant differences in other clinical characteristics, such as resectability status, CA 19-9 levels, and histologic type, between those with and without favorable PR.

**Table 1:** Patient characteristics Favorable PR patients demonstrated higher ypT1/T2 prevalence in both training (*P* = 0.04) and testing (*P* = 0.04) sets. Moreover, a significant increase in ypN0 was observed in the testing set among those with favorable PR (*P* = 0.04). PR, pathological response; BMI, body mass index; GnPO+ITC, gemcitabine+nab-paclitaxel+oxaliplatin+itraconazole; FOLFIRINOX, fluorouracil+leucovorin+irinotecan+oxaliplatin; GEM, gemcitabine; nP, nab-paclitaxel; well, well differentiated adenocarcinoma; mod, moderately differentiated adenocarcinoma; por, poorly differentiated adenocarcinoma

Characteristics	Training cohort			Testing cohort		
	With favorable PR	Without favorable PR	P	With favorable PR	Without favorable PR	P
	n = 29	n = 31		n = 12	n = 14	
Age (year)	64.0 (39-83)	68.0 (44-82)	0.98	71.5 (51-80)	64.5 (42-78)	0.17
Sex, n (%)			0.61			0.70
Men	14 (48.3)	18 (58.1)		5 (41.7)	8 (57.1)	
Women	15 (51.7)	13 (41.9)		7 (58.3)	6 (42.9)	
BMI (kg/m^2^)	20.5 (14.5-27.7)	22.1 (16.1-31.0)	0.09	21.9 (18.3-29.4)	20.5 (16.6-32.3)	0.52
Tumor location, n (%)			0.45			1.00
Head	18 (62.1)	16 (51.6)		7 (58.3)	9 (64.3)	
Body/Tail	11 (37.9)	15 (48.4)		5 (41.7)	5 (35.7)	
Resectability status, n (%)			0.77			0.86
Resectable	9 (31.0)	13 (41.9)		4 (33.3)	4 (28.6)	
Borderline resectable	4 (13.8)	4 (12.9)		1 (8.3)	3 (21.4)	
Unresectable (locally advanced)	11 (37.9)	8 (25.8)		4 (33.3)	4 (28.6)	
Unresectable (metastatic)	5 (17.2)	6 (19.4)		3 (25.0)	3 (21.4)	
Pre CA 19-9 (U/ml)	230 (0-2719)	165 (0-29157)	0.60	236.5 (15-6217)	171.5 (7-29161)	0.68
Post CA19-9 (U/ml)	49 (0-3877)	138 (0-1669)	0.20	71 (6-115)	54 (0-3309)	0.90
Pancreatectomy, n (%)			0.29			1.00
Proximal	17 (58.6)	13 (41.9)		7 (58.3)	9 (64.3)	
Distal	11 (37.9)	14 (45.2)		5 (41.7)	5 (35.7)	
Total	1 (3.4)	4 (12.9)		0 (0)	0 (0)	
Venous resection, n (%)	10 (34.5)	6 (19.4)	0.25	3 (25.0)	4 (28.6)	1.00
Arterial resection, n (%)	4 (13.8)	1 (3.2)	0.19	1 (8.3)	2 (14.3)	1.00
Chemotherapy, n (%)			0.06			0.10
GnPO-ITC	20 (69.0)	13 (41.9)		6 (50.0)	9 (64.3)	
FOLFIRINOX	0 (0)	0 (0)		2 (16.7)	0 (0)	
GEM + nP	0 (0)	2 (6.5)		0 (0)	3 (21.4)	
GEM + S-1	9 (31.0)	16 (51.6)		4 (33.3)	2 (14.3)	
Chemo duration (months)	5.0 (0.7-10.1)	2.1 (0.7-21.9)	0.42	6.75 (1.2-12.5)	6.35 (1.5-8.2)	0.57
Histologic type, n (%)			0.62			0.83
Well/Mod	26 (89.7)	25 (80.6)		9 (75.0)	11 (78.6)	
Por	3 (10.3)	4 (12.9)		1 (8.3)	2 (14.3)	
Adenosquamous	0 (0)	2 (6.5)		2 (16.7)	1 (7.1)	
ypT stage, n (%)			0.04			0.04
T1/T2	11 (37.9)	4 (12.9)		7 (58.3)	2 (14.3)	
T3/T4	18 (62.1)	27 (87.1)		5 (41.7)	12 (85.7)	
ypN stage, n (%)			0.79			0.04
N0	13 (44.8)	12 (38.7)		8 (66.7)	3 (21.4)	
N1	16 (55.2)	19 (61.3)		4 (33.3)	11 (78.6)	

Radiomic feature extraction and selection

**Figure 2:**
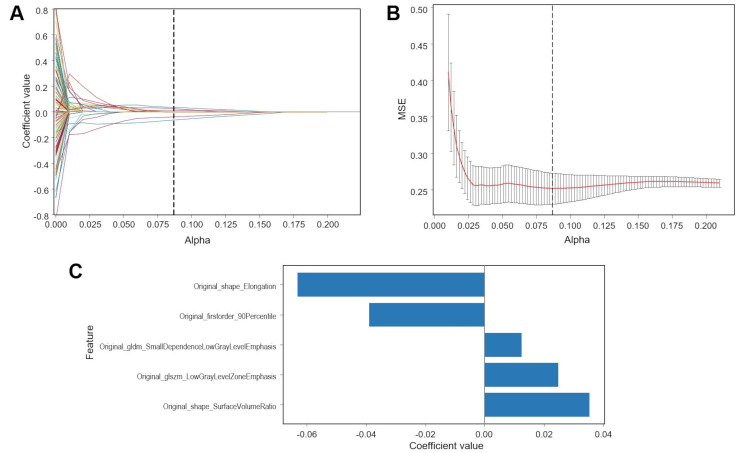
Radiomic feature selection (A) Coefficient curve depicting changes in the regularization parameter (alpha) during the utilization of Lasso logistic regression for radiomic feature selection. (B) Alpha selection in LASSO logistic regression with minimum MSE. (C) Coefficient values corresponding to the selected radiomic features. LASSO, least absolute shrinkage and selection operator; MSE, mean squared error; GLDM, gray level dependence matrix; GLSZM, gray level size zone matrix.

A total of 123 radiomic features were extracted from the CT images of each patient using PyRadiomics. A training set of 60 patients was used for LASSO logistic regression, and five features that were predictive of a favorable PR were selected based on the best alpha identified through cross-validation (Figure 2A, 2B). These included one first-order feature, two 3D shape-based features, one GLDM feature, and one GLSZM feature. The coefficient values for the selected features are presented in Figure 2C.

ML model construction and validation

**Figure 3:**
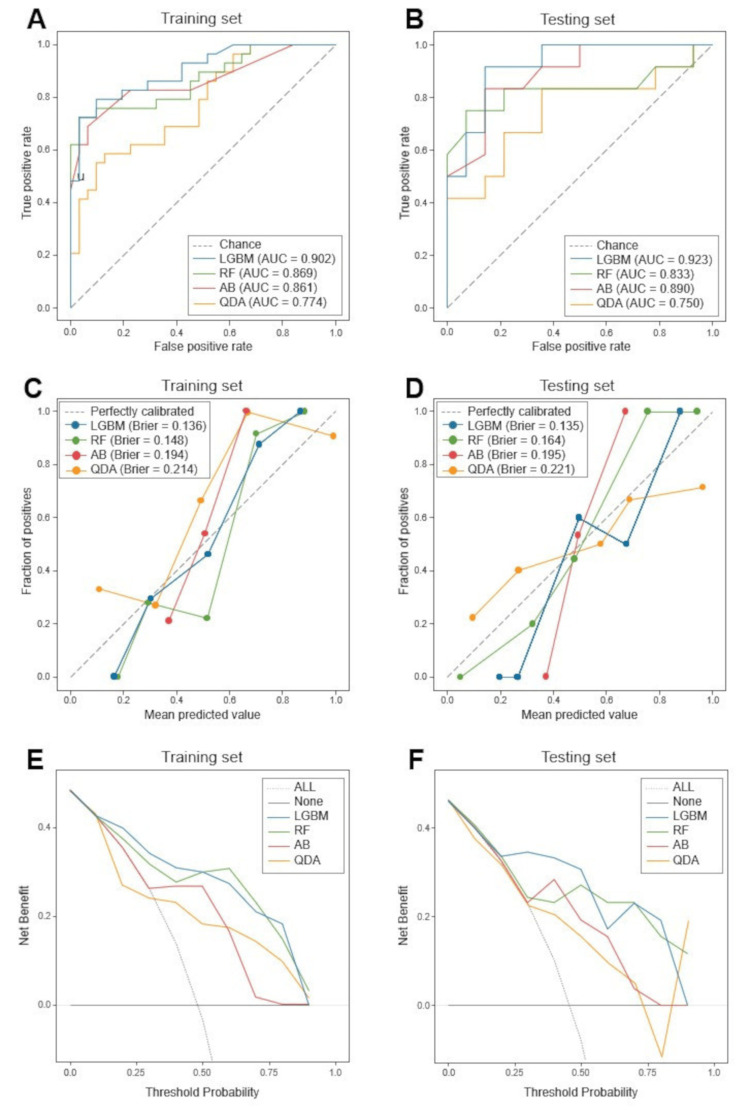
ROC, calibration, and decision curve analyses (A, B) ROC curves for predicting favorable pathological responses in the training (A) and testing (B) sets. (C, D) Calibration curves demonstrating the reliability and calibration performance of the models in the training (C) and testing (D) sets. (E, F) Decision curve analysis demonstrating the utility of machine learning models in the training (E) and testing (F) sets. ROC, Receiver operating characteristic; LGBM, Light Gradient Boosting Machine; RF, Random Forest; AB, AdaBoost; QDA, Quadratic Discriminant Analysis.

We used four classifiers (LGBM, RF, AB, and QDA) to construct radiomics-based models. In the training set, LGBM had the highest AUC (0.902), followed by RF (0.869), AB (0.861), and QDA (0.774) (Figure 3A). Brier scores were consistent with this order, with LGBM achieving the lowest score (0.136), followed by RF (0.148), AB (0.194), and QDA (0.214) (Figure 3C). The decision curve analysis showed that LGBM and RF had favorable performance, while AB and QDA had inferior performance (Figure 3E). In the testing set, LGBM maintained the highest AUC (0.923), followed by AB (0.890), RF (0.833), and QDA (0.750) (Figure 3B). Brier scores revealed a nuanced variation, with LGBM achieving the lowest score (0.135), followed by RF (0.164), AB (0.195), and QDA (0.221) (Figure 3D). The DCA for the testing set consistently depicted LGBM in a favorable position compared to other models (Figure 3F). Additional metrics outlining the predictive ability of the classifiers are presented in Table 2. Overall, the LGBM model outperformed the other classifiers in terms of both AUC and Brier scores, underscoring its superior performance, as supported by the DCA.

**Table 2:** Performance of models AUC, Area under the curve; LGBM, Light Gradient Boosting Machine; RF, Random Forest; AB, AdaBoost; QDA, Quadratic Discriminant Analysis

Algorithm	Data	AUC	Brier score	Accuracy	Sensitivity	Specificity	f1 score
LGBM	Training	0.902	0.136	0.817	0.793	0.839	0.807
	Testing	0.923	0.135	0.846	0.833	0.857	0.833
RF	Training	0.869	0.148	0.817	0.759	0.871	0.800
	Testing	0.833	0.164	0.808	0.583	1.000	0.737
AB	Training	0.861	0.194	0.783	0.621	0.936	0.735
	Testing	0.890	0.195	0.731	0.583	0.857	0.737
QDA	Training	0.774	0.214	0.700	0.448	0.936	0.591
	Testing	0.750	0.221	0.692	0.667	0.714	0.667

## Discussion

In general, patients with PDAC undergoing neoadjuvant/conversion chemotherapy are considered for surgery unless they exhibit local tumor progression, new metastases, or a significant decline in performance status. However, many patients who undergo subsequent surgery experience early disease recurrence and shortened overall survival time [25]. While there are multiple factors contributing to this poor outcome, the two primary challenges are the difficulty of identifying occult metastases, especially in the liver, and the evaluation of PR before surgery. In an analysis of 223 patients with PDAC who underwent neoadjuvant chemoradiation and subsequent pancreaticoduodenectomy, Chatterjee et al. reported significantly better overall survival in patients with Evans grade III or IV tumors compared to those with grades I, IIa, and IIb [17]. In addition, some researchers demonstrated that PR with Evans grade IIb or greater was associated with more favorable survival outcomes compared to grade I or IIa [18-20]. Given that PR can be a surrogate marker for systemic disease control and is associated with survival, accurate preoperative PR assessment can guide the selection of optimal surgical candidates and the timing of surgery, ultimately improving long-term outcomes.

In this study, we analyzed post-chemotherapy CT images of PDAC to extract radiomic features related to favorable PR. Among the five selected radiomics features, 'original_shape_Elongation,' 'original_firstorder_90Percentile,' and 'original_shape_SurfaceVolumeRatio' demonstrated larger coefficients in the LASSO regression compared to the other texture-based features. This suggests the potential of first-order statistical or shape-based features to capture PR more effectively than texture-based features, although such effectiveness may be influenced by the dataset and analysis method. Subsequently, the LGBM model demonstrated good performance with an AUC exceeding 0.9 and a Brier score below 0.15 in both the training (AUC: 0.902, Brier score: 0.136) and the testing (AUC: 0.923, Brier score: 0.135) sets, indicating effective results. In addition, DCA, estimating the clinical utility of the model, revealed that it provided more benefits compared to both the treat-all and treat-none schemes. LGBM is a fast, distributed, high-performance gradient-boosting framework based on a decision tree algorithm [26]. The reason for the superior performance of LGBM in the current study was unclear, but it might be algorithmically attributed to its ability to learn non-linear relationships, its robustness to overfitting, and its less sensitivity to the amount of training data [27].

To date, only a limited number of studies have explored CT radiomics for PR assessment in PDAC. In a study by Chen et al., involving 20 patients with pancreatic head cancer, changes in mean histograms of CT numbers, standard deviation, skewness, and kurtosis were associated with PR as defined by the College of American Pathologists score [28]. Borhani et al., who investigated the correlation between CT-derived texture features and PR in 39 patients with PDAC, showed that patients with higher mean positive pixels at pretreatment CT were more likely to have favorable PR of Evans grade IIb or greater [20]. In 24 patients with either resectable or borderline resectable pancreatic head cancer, Nasief et al. demonstrated that, while the AUC for predicting PR was 0.69 with CA 19-9 alone, it improved to 0.87 when combining CA 19-9 with delta radiomic features obtained from daily CT-guided chemoradiotherapy [29]. In their previous report, the authors had shown that delta radiomic features, integrated into an ML model (Bayesian-regularization-neural-network), achieved an AUC of 0.94 for predicting PR in resectable PDAC [30]. To our knowledge, our study represents the second investigation using ML for a CT radiomics-based PR prediction model in PDAC. Moreover, it is the first study to validate the performance of multiple ML algorithms using post-chemotherapy CT images exclusively.

There were several limitations in our study. Firstly, the sample size was limited, and the absence of an external validation cohort hinders the broader applicability of our findings. Secondly, the cohort exhibited heterogeneity, particularly in terms of resectability status and diverse regimens of chemotherapy, introducing complexities in result interpretation. Thirdly, using only one radiologist for tumor segmentation and one pathologist for assigning Evans grades may introduce limitations, as the absence of multiple evaluators could affect the robustness of our findings. Another limitation is that we did not utilize pre-chemotherapy CT data. Predicting PR before treatment initiation could influence the choice of upfront surgery over chemotherapy, especially in resectable PDAC. Finally, inherent challenges associated with radiomics, such as the time-consuming segmentation process and data analysis, should be acknowledged.

## Conclusions

This study demonstrated the efficacy of the radiomics-based ML model, particularly the LGBM algorithm, for predicting PR in patients with PDAC undergoing preoperative chemotherapy. The model shows promise in optimizing patient selection for surgery and surgical timing, leading to personalized treatment strategies. Despite this encouraging result, further research with larger datasets and external validation is essential to refine and solidify the clinical application of this radiomics approach for PDAC.
